
JOURNAL INFORMATION
==============================
NLM Title Abbreviation: ScientificWorldJournal
Journal ID: ScientificWorldJournal
NLM Journal ID: 101131163
Journal ID: TSWJ

The Scientific World Journal

ISSN: 2356-6140
EISSN: 1537-744X
Publisher: Wiley

ARTICLE INFORMATION
==============================
PMCID: PMC6009729
PMID: 29973796
DOI: 10.1100/tsw.2002.22
Article ID: 873048
Article version: 1
Subjects: Short Report

Combining Bioinformatics and Biophysics to Understand Protein-Protein and Protein-Ligand Interactions

Honig Barry *
Howard Hughes Medical Institute, Department of Biochemistry and Molecular Biophysics, Columbia University, 630 W. 168 St., New York, NY 10032, USA
*Barry Honig: bh6@columbia.edu
Publication date: 2002
Electronic publication date: 2002 Apr 24
Volume: 2
First page: 43
Last page: 44
Copyright: Copyright © 2002 Barry�Honig.
Copyright year: 2002
License: This is an open access article distributed under the Creative Commons Attribution License, which permits unrestricted use, distribution, and reproduction in any medium, provided the original work is properly cited.
License URL: https://creativecommons.org/licenses/by/3.0/

Funding: European Commission GM30518 National Science Foundation MCB-9808902 National Science Foundation DBI-9904841

==============================
Advances in Gene Technology: The Genome and Beyond –
Structural Biology for Medicine (Proceedings of the 2002 Miami
*Nature Biotechnology* Winter Symposium)
*TheScientificWorld* 2002, 2(S2), 43–44
ISSN 1532-2246; DOI 10.1100/tsw.2002.22

### COMBINING BIOINFORMATICS AND BIOPHYSICS TO UNDERSTAND PROTEIN-PROTEIN AND PROTEIN-LIGAND INTERACTIONS

Barry Honig
Howard Hughes Medical Institute, Department of Biochemistry and Molecular Biophysics,
Columbia University, 630 W. 168 St., New York, NY 10032
bh6@columbia.edu

**INTRODUCTION.** The increasing numbers of proteins whose three-dimensional structures have been determined will have major impact on the ability to exploit genomic data. Sequence alignments will become more meaningful, protein structure prediction will become more accurate, and the prediction of protein function will become increasingly refined and precise. Such developments will require that sequence, structure, and physical chemical information be fully integrated and correlated with biological data in as much detail as possible. We have been developing a series of computational tools with the goal of detecting relationships among amino acid sequence, protein structure and protein function. In this context, recent computational advances in using structure to improve sequence alignments, in homology model building and in the calculation of binding affinities will be summarized as will their combined use, with specific application to understanding the principles of protein-protein and protein-ligand interactions.

**METHODS.** Our basic approach involves calculating protein folding and binding free energies as well as contributions of individual amino acids to these free energies, and correlating these energetic contributions with sequence patterns and with physical and chemical properties of the protein. With regard to binding, we are particularly interested in delineating features that may dictate specificity vs. affinity and in predicting binding specificity from sequence alone. Binding free energies are described in terms of electrostatic and hydrophobic interactions. The former are calculated using finite difference Poisson-Boltzmann methods while the latter are generally calculated from free energy-surface area relationships.

**RESULTS AND DISCUSSIONS.** Our approach to the calculation of binding free energies is validated by its ability to select the correct conformation of protein-protein complexes from a large number of alternative complex geometries. The factors that determine binding free energies will be discussed with particular emphasis on understanding how protein interfaces are designed, in a structural sense, to exploit different combinations of electrostatic and hydrophobic interactions to achieve both affinity and specificity. This information is derived in part by calculating binding free energies for different interfaces and correlating these with structural features on the interface and with sequence patterns. In addition, we have created a database of all protein-protein interfaces of known structure. This allows us to carry out statistical analysis of different interfaces which, when combined with binding free energy calculations, provides a detailed picture of how protein surfaces are designed to effect different functions and exhibit a wide range of binding specificities.

An important feature in any attempt to compare the properties of different proteins is to obtain an accurate sequence alignment and, when possible, an accurate structure alignment as well. We have developed a novel approach to use structural information to improve sequence alignment and detection. First, multiple sequence alignments are generated from sequences that are closely related to each sequence of known three-dimensional structure in a particular protein family. The alignments generated in this way are then merged through a multiple structure alignment of all family members of known structure. The merged alignment is then used to generate a Hidden Markov Model (HMM) for the family in question. The HMM generated can be used to search for new family members or to improve alignments for distantly related family members that have already been identified. Application of a profile generated for SH2 domains indicates that the Janus family of nonreceptor Protein Tyrosine Kinases (JAKs), contain SH2 domains. Homology modeling and the use of electrostatic methods to calculate the pKa's of the normally conserved arginine at the key phosphotyrosine binding position in SH2 domains suggest that one of the JAKS, TYK2, may contain a domain with an SH2 fold that has a modified binding specificity. This example demonstrates how a combination of theoretical methods can generate novel biological insights.

Our approach to predicting specificity from sequence alone requires that we first determine the binding determinants of complexes of known three-dimensional structure. Using SH2 domains as an example, we calculate the free energy contribution to binding of every residue in the protein for every SH2 domain-peptide complexes of known structure. For each complex we identify the residues that provide the most significant energetic contribution to binding and then map these onto our sequence profile of SH2 domains. A new sequence of unknown specificity is then aligned to this profile thus allowing us to associate sequences of unknown specificity to one of the structurally characterized complexes and, in this way, to predict the binding specificity of a large number of the sequences. Our predictions are in excellent agreement with experiment.

With regard to structure prediction, we have made a number advances that we believe will significantly improve the accuracy of homology modeling. Specifically, we have succeeded in predicting the conformation of buried side chains to close to experimental accuracy, in obtaining extremely high loop prediction accuracies and in refining modeled structures so as to more closely resemble the correct structure than the original template. In addition, the new alignment procedure described above, and our biophysical studies of folding free energies have led to the development of a new and accurate procedure to evaluate homology models. These and other developments have been incorporated into new homology modeling software that will be described.

**ACKNOWLEDGEMENT.** This work was supported by the NIH GM30518, NSF grants MCB-9808902 and DBI-9904841.

**REFERENCES**

1. Yang, A.-S. and Honig, B. (2000) J. Mol. Biol. 301, 665–678.
2. Yang, A.-S. and Honig, B. (2000) J. Mol. Biol. 301, 679–689.
3. Yang, A.-S. and Honig, B. (2000) J. Mol. Biol. 301, 691–711.

4. Xiang, Z. and Honig, B. (2001) J. Mol. Biol. 311, 421–430.
5. Norel, R., Sheinerman, F., Petrey, D., and Honig, B. (2001) Prot. Sci., in press.
6. Al-Lazikani, B., Sheinerman, F., and Honig, B. (2001) Proc. Natl. Acad. Sci., in press.
